# Comparison of Elicitation Approaches in Early Stage HTA Applied on Artificial Thymus for Patients with DiGeorge Syndrome

**DOI:** 10.3390/healthcare11223002

**Published:** 2023-11-20

**Authors:** Marija Gorelova, Karolina Rysankova, Gleb Donin, Peter Kneppo, Vladimir Rogalewicz

**Affiliations:** Department of Biomedical Technology, Faculty of Biomedical Engineering, Czech Technical University in Prague, 272 01 Kladno, Czech Republic

**Keywords:** elicitation, expert opinion, early health technology assessment, eHTA, artificial thymus

## Abstract

This paper focuses on research in expert elicitation as a part of the early stage health technology assessment (eHTA). The current state of affairs is analysed and two elicitation approaches are compared—the four fixed intervals method and the histogram method—as applied to an example of early assessment of clinical effectiveness of artificial thymus for patients with DiGeorge syndrome. A survey was carried out consisting of four questions concerning the topic, with the aim to apply the elicitation methods. Eight experts answered the questions using both elicitation methods. Based on their answers, the methods were compared visually and by means of statistical tests. In order to compare the perception of the two elicitation methods, the survey also included questions regarding the experts’ subjective preferences. The results of the comparison of the two elicitation approaches did not clearly confirm which method was more beneficial and better; however, it was possible to indicate which of the two methods is better suited for different types of experts. Before selecting an elicitation method as a part of eHTA, it is advisable to effectively consider the technology to be assessed and the type of experts to be invited to share their opinion.

## 1. Introduction

Medical devices have a short life cycle, which is why a lot of research has been recently carried out to find methods for assessing medical devices already in the early phase of development. Conducting a health technology assessment (HTA) even before clinical testing and a market launch should improve the investment targeting, stimulate development, and thus save on the cost of clinical tests that a new technology might fail. The results of this process are useful for decision making regarding the future direction of research, setting minimum effectiveness thresholds, supporting pricing, and payment decisions [1].

This sort of assessment is called “early (stage) HTA” (eHTA). Early stage HTA is an assessment of health technologies at the stage of research and development. It is most commonly used as a tool for identifying economic determinants of individual health technologies. Early stage models can be used to inform the design and management stage of health technologies under development in order to mitigate the risks associated with placing the technology on the market and its inclusion into the public insurance reimbursement systems. eHTA, combined with early health economics modelling, has been increasingly used by manufacturers as an approach to identifying the added value of new health technologies. The results of such a process are useful for the following:

- Deciding on the next direction of development;
- Setting minimum efficiency limits for a new technology compared with the currently available comparators;
- Providing support for pricing and payment settings [2,3].

An analysis of several large-scale theoretical studies showed that, in general, the eHTA process for medical devices consists of three stages:

1. Conceptual modelling of use of the new medical technology;
2. Determination of unknown model parameters by an expert elicitation;
3. Analysis of commercial options for the new medical technology under assessment [2,3].

The first phase, modelling, is used for new medical equipment for which the data are neither known nor obtainable. In that case, data are simulated using data on similar older medical devices. If some data essential for assessment are still missing (in case of a newly developed equipment), the process moves to the elicitation phase. Elicitation is a method that can be used to obtain the probability distributions of unknown values based on expert opinions. For example, elicitation identifies the opinion of experts about the treatment outcome or improving the quality of life. It is a process of formal elicitation of expert opinions used in decision-analytic models in healthcare [1,2,3,4,5,6].

The recent development in healthcare research has confirmed the benefits of elicitation as a form of expert opinion on as-yet unknown effects in a probabilistic form [1,5,7]. These methods can be used to characterize the parameters of uncertainty. However, there are no unified guidelines, rules, or prescribed national or general procedures for conducting an eHTA study. Furthermore, the elicitation process is the least described part of eHTA in published studies. There are a lack of recommendations and requirements regarding the suitability of selection of an elicitation method for eHTA. It can be assumed that the observation of graphic and numerical methods can be completely different depending on the type of expert, their profession, and their experience with similar investigations [3].

Grigore et al. [8] presented an overview of several eliciting methods for healthcare studies and their comparison. They stated that the main priority for correctly obtaining estimates is the selection of experts. Ideally, they should be professionals with the most extensive experience and knowledge in the particular area. According to the authors, very few medical studies address the number of experts that should provide their opinions, Grigore et al. [8], however, suggested that 6 to 12 experts should suffice in most cases. In their study, the respondents—mostly physicians specializing in oncology or urology—were asked to express their opinions in the form of a probability distribution using two elicitation methods: the histogram method and the hybrid method. Both methods proved to be equally useful, while it was impossible to unequivocally determine which one was better. However, the experts reported that the histogram method was easier to use as it allowed for graphic elicitation. It is a discrete form of the probability density function, where the expert was presented with a diagram and asked to place a fixed number of crosses on it according to their professional opinion. The result of this method is a histogram [8].

The topics of cost-effectiveness modelling and the related elicitation of expert opinions were described in general terms by Bojke et al. [9], who described elicitation as a process of expert knowledge transformation, which provides information that can be compared with the collection of experimental evidence. However, elicitation is a relatively low-cost source of evidence compared with the collection of experimental evidence. The authors also attempted to define an expert whose opinion should be sought. In general, they agreed that participants should be reputable specialists in the particular field. One of the methods they mentioned and discussed was the fixed intervals method, which presents experts with a set of intervals and the experts are asked to add a probability value to each interval. Another method they mentioned is the histogram method. This method is similar in principle, but enables elicitation in graphic form, where, for example, a form is presented to an expert and they are asked to place a fixed number of crosses on it, according to their expert opinion. Expert respondents have concluded that the histogram method is very simple to use, even for non-technically-oriented experts, while the fixed intervals method is, in their opinion, more precise. 

The above-mentioned elicitation methods use different procedures, and, thus, before launching an eHTA study, one should always ask “which method should be used” and “whether the choice of the method would affect the result”. The main aim of our study is, therefore, to analyse and compare the four fixed intervals method and the histogram method of the expert opinion elicitation as a part of eHTA in a specific model case. Both elicitation approaches are de facto methods with fixed intervals, but with different granularity. However, the histogram method is a graphical−visual method, unlike the four fixed intervals method, which is a numerical method. This research is a survey of how experts perceive these two methods, with the aim to identify possible differences between the answers using individual methods—graphical and numerical methods. To illustrate this and to obtain relevant data, we needed to choose a completely new potential healthcare technology in the early stages of development. The technology should be in a completely hypothetical state regarding use on patients, but, at the same time, should have a sufficiently detailed description available for experts to familiarize themselves with it. We selected a medical technology from the field of tissue engineering that was still in the development phase—artificial thymus [10,11]. It is primarily intended for the treatment of DiGeorge syndrome, but potentially may also be used for other T-lymphocyte production disorders such as leukaemia, autoimmune diseases, and AIDS [12,13,14,15].

Artificial thymus technology has not been clinically tested or tried yet, and, hence, most of its effects are unknown. The technology is currently in the design and development stage. So far, the artificial thymus has been tested only on laboratory mice, but with positive results [10,11,12,16,17,18,19]. For an eHTA study, the as-yet unknown technology effect parameters could be, for the time being, substituted by expert opinions obtained through an elicitation method. 

The modelled elicitation process could realistically be used to determine unknown parameters in eHTA study of new and modern technologies that are untested on human patients, such as the artificial thymus. In this study, we investigated using a staged elicitation of how experts perceive individual approaches and to identify possible differences between answers using individual methods—graphical and numerical.

The objective of this study was to compare how medical experts managed two seemingly similar elicitation procedures recommended for utilization in investigations of healthcare problems, including eHTA. Based on the broad expertise of scientists at the Faculty of Biomedical Engineering of the Czech Technical University in Prague, the artificial thymus was selected for the case study as a technology not yet used in human medical research. It was considered to be a good and typical representative of any medical device that will proceed to the development phase in near future [3].

## 2. Materials and Methods

In order to achieve the goals of this study, to compare two elicitation methods, conceptual decision-analytic models were first developed based on an analysis of transplantations in complete DiGeorge syndrome cases [10,11,12,14,15,16,18].

The unknown technology effect parameters were chosen based on a conceptual model. These were the percentage of patients referred for the new treatment, the success rate of the artificial thymus treatment, and the mortality rate of this treatment. To find the values of these parameters, experts were asked three questions. The last, fourth question was a “reference” question, the answer to which we already knew from published literature. This question was added to confirm that the respondents really were experts on the topic. A reference question can also be a useful tool for weighting the experts for a following aggregation (e.g., using the Classical or Cooke’s Method) and application-elicited estimates in an eHTA model [20,21]. Published studies indicated that the survival rate after transplantation in patients with complete DiGeorge syndrome is 73% (SD = 2.12, CI = 2.08, α = 0.05) [12,15,22].

The four questions asked were as follows:

1. What percentage of patients with complete DiGeorge syndrome would be eligible candidates for the artificial thymus treatment?
2. In your opinion, what percentage of patients with complete DiGeorge syndrome could see improvements in their health state after an application of the artificial thymus?
3. In your opinion, in what percentage of cases the application of an artificial thymus can result in patient’s death?
4. What is the current survival rate after transplantation in patients with complete DiGeorge syndrome?

Experts answered all of the questions, subsequently using both selected elicitation methods, by receiving a form. The questionnaire contained a table that was filled in by the experts when they answered questions using the histogram method. This provided a visual representation of the expert opinion probability distribution. All of the answers were entered into a table in the form of 20 crosses, where each cross represented a 5% probability that the real value would be in the respective interval. All crosses in one table needed to equal 100%. The horizontal axis represented the relative population size and the width of marked area reflected certainty: the lower the certainty of the expert’s opinion, the wider the marked area. The vertical axis represented the probability of the effect: the higher the probability assigned by the expert to the respective interval, the higher the marked area.

For answers by the four fixed intervals method, the questionnaire contained a much simpler table. Unlike the histogram method, the answers were not required in the form of a graphic representation, but by numerical values. A specific probability value (in percentages) was assigned to each 25% interval. For each question, the probabilities needed to equal 100%.

We created a questionnaire (see Supplementary Materials S1 for the English translation of the whole questionnaire) consisting of a description of the topic to be assessed and instructions that provided a simple explanation of the principle of answering in the form of a probability distribution. The next part included four questions regarding the assessed topic (see above).

At the end, the questionnaire included questions about the expert’s subjective preferences and opinions on both elicitation methods. These subjective opinions were collected in order to compare the elicitation methods from the point of view of their demands and comprehensibility. The first question asked whether the respondents had already taken part in any expert opinion solicitation (YES or NO). The second question asked the participants to subjectively judge their own statistical skills (basic, good, or excellent). The third question asked to indicate on a scale of 1 to 5 (1 = very difficult, 5 = very easy) how difficult it was for the participant to complete the current survey. The last two questions asked the participants which elicitation method they considered more understandable and/or reliable. Finally, the questionnaire provided space for respondents’ comments.

The choice of relevant experts—specialists in the particular field, in this case immunology—was crucial to find reliable answers to our questions. Emails were sent to 15 leading immunological departments of university hospitals to find the most experienced experts. Each hospital nominated approximately two suitable experts (a clinician and a lab technician), and recommended them for participation in the research.

The main criterion for the selection of experts from clinicians was the length of their professional career. As physicians first go through the attestation process in a specific field after their graduation from medical school, we set the minimum length of practice at 12 years. Another criterion was at least two internships or residencies abroad and, due to the fast development in immunology, regular participation in international conferences. The study also employed immunology lab technicians who had completed at least a Master’s education specialising in immunology, had been involved in research projects, and were familiar with DiGeorge syndrome. Care was taken to ensure the impartiality of the experts, so that they were not related to the evaluated technology and were not part of a professional team involved in the development of this technology.

A total of 26 experts were nominated by departments of university hospitals—10 clinicians and 16 lab technicians. After contacting individual experts, it was found that only eight clinicians and six lab technicians met all the required criteria stated in the invitation letter. Some of the compliant experts then excused themselves due to lack of time.

Based on the above, four clinicians from immunology departments and four immunology lab technicians were selected and agreed to participate in the survey. The survey took place as a face-to-face meeting and each expert received a paper form of questionnaire. Before responding to the questionnaire, the respondents received instructions in an oral form, including an introduction to the topic, an explanation of the elicitation methods, and a presentation of the overall aims of the research project. The instructions took approximately 15 min. The respondents were then presented with the questionnaire on paper. While filling in the questionnaire, the experts were allowed to ask additional questions if any of the questions were not entirely clear. It took respondents 30 min on average to finish the survey.

During the elicitation exercise, a consistency check was performed so that the total probability from all intervals reached 100% and did not exceed it.

The answers to each question obtained by both elicitation methods were subjected to Pearson’s χ^2^ test of homogeneity to find whether the samples could be considered to be taken from the same distribution. Thus, we tested the similarity or divergence of the results from both elicitation methods. Answers to questions regarding the experts’ subjective preferences were entered into tables and are represented in pie charts in this paper. The purpose of these questions was to explore participants’ opinions about which method is easier to understand and follow.

## 3. Results

All of the doctors and lab technicians invited to participate in the project answered all questions in the questionnaire. A consistency check of the experts’ responses confirmed that the overall probability from all intervals was 100%. No errors were found.

**Figure 1:**
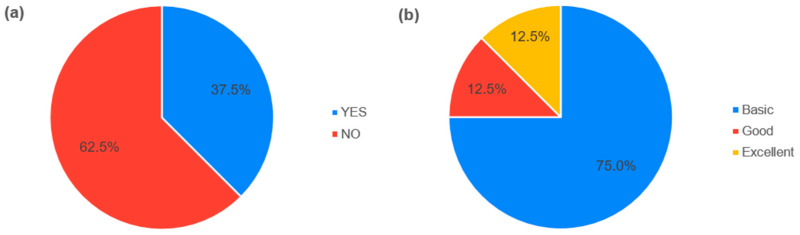
Expert’s skills. (**a**) Answers to the question about participation in a research of expert opinions. (**b**) Answers to the question about the statistical skills.

From the survey about previous skills of experts, it was found that five experts had no previous experience in asking for expert opinion. Three experts already had experience with elicitation (Figure 1a). The experts subjectively judged their statistical skills as follows: six experts chose “basic”, one expert “good” and one expert “excellent” level (Figure 1b).

**Figure 2:**
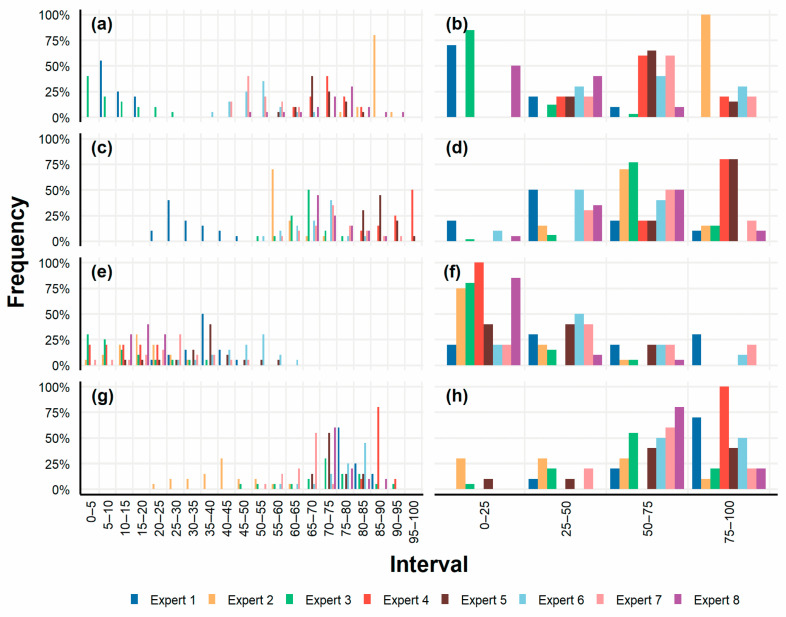
Answers obtained by the by the histogram (**a**,**c**,**e**,**g**) and by the four fixed intervals (**b**,**d**,**f**,**h**) elicitation methods. (**a**,**b**) Answers to the first question about percentage of patients with complete DiGeorge syndrome who could be eligible candidates for the artificial thymus treatment. (**c**,**d**) Answers to the second question about the percentage of patients with complete DiGeorge syndrome who could see improvements in their health state after an application of the artificial thymus. (**e**,**f**) Answers to the third question about possible mortality due to the application of the artificial thymus. (**g**,**h**) Answers to the fourth “reference” question about the current survival rate after the transplantation in patients with complete DiGeorge syndrome.

The experts’ answers about complete DiGeorge syndrome are presented in a graphic form in this paper (for the whole data set see Supplementary Materials S2). Figure 2a shows the answers to the first question (percentage of patients with complete DiGeorge syndrome who could be eligible candidates for artificial thymus treatment) by the histogram method and it clearly indicates that Expert 1 and Expert 3 differed in their answers from all of the other experts. Based on the elicitation method principles, the distribution range of probabilistic answers represented certainty. The narrower the interval of the population range, the more certain the expert was in their opinion regarding the given question. Expert 1 was very certain in their answer to the first question and distributed their 100% of probability only among three intervals in the lower value range. Their answer indicates that there was only a small percentage of DiGeorge syndrome patients with a high probability of being eligible for the artificial thymus treatment. Expert 3 provided a similar answer but at a lower certainty, distributing their opinion over six intervals. In contrast with these two respondents, Experts 2, 4, 5, 6, 7, and 8 placed their answers closer to a higher percentage of eligible patients. Expert 2 was the most certain in their opinion. Expert 8 provided an answer with the smallest certainty of opinion and the biggest range. From the elicitation method perspective, we could assume that the correct answer lay in intervals that accumulated the biggest share of probability assigned by all of the experts.

Figure 2b shows the answers to the same question from the four fixed intervals method. If we visually compare Figure 2a,b, we find that the probability distribution across intervals corresponded, in most cases, to the answers from the histogram method. Expert 8 was the only one whose answer was different from the one provided in the histogram method, now assigning the highest probability to the 0–25% interval. Answers from the two methods were also compared using Pearson’s χ^2^ test. Test results for the first question did not reject the zero hypothesis (*p* = 0.3965), i.e., we could not reject the possibility that the answers from both elicitation methods did not differ from each other.

Figure 2c shows answers to the second question (the percentage of patients with complete DiGeorge syndrome who could see improvements in their health state after an application of the artificial thymus) from the histogram method. Once again, Expert 1 stood out from the other respondents and, unlike the others, provided a very sceptical opinion. Their answer can be paraphrased as follows: “I think that the highest probability of health improvement in 25–30% patients with complete DiGeorge syndrome is 40%”. On the other hand, the biggest optimists in this respect were Experts 4 and 5, who assigned probability to the last four intervals, i.e., in the 80–100% range. Experts 2, 3, 6, 7, and 8 distributed probability with varying certainty levels in similar intervals in the 50–90% range.

Figure 2d shows answers to the same question from the four fixed intervals method. There were several differences compared with the histogram method. Despite the different answers, we can conclude that, in the experts’ subjective opinion, the highest probability of health improvement after the artificial thymus application could be in 65–100% of patients. The statistical test for this question rejected the zero hypothesis (*p* = 0.0069), i.e., the answers provided by the two elicitation methods differed.

We can see an increasing uniformity of expert answers with each subsequent question. If, for the first question, the answers from the histogram method were quite diverse and covered almost the entire set of intervals (Figure 2a), then for the third question, we observed a reduction in the set of intervals used by almost half (Figure 2e). This was also evident in the four fixed intervals method, although not as pronounced. Figure 2e shows answers to the third question (possible mortality due to the application of the artificial thymus) from the histogram method. Most experts answered with a high level of uncertainty. The probability was distributed over the first half of the interval range in most answers. The answers to the same question for the four fixed intervals method (Figure 2f), again, partly diverged from the answers from the histogram method, especially the answers given by Experts 1, 6, and 7, who assigned probability to the higher-value intervals. Pearson’s χ^2^ test rejected the zero hypothesis (*p* = 0.00030); hence, the elicitation methods yielded different answers. The differences were already apparent from the graphic representation.

The fourth question (the current survival rate after the transplantation in patients with complete DiGeorge syndrome) was included as a “reference” question. The answers obtained by the elicitation methods are depicted in Figure 2g,h. They indicated whether the respondents were real experts with in-depth knowledge concerning DiGeorge syndrome. The published research suggested that the correct answer was the survival rate of 73% (SD = 2.12, CI = 2.08, α = 0.05) [12,15,16,22]. According to Figure 2g, with the results from the histogram method, the closest answer to the 73% value was that of Expert 5. Other experts that came close to the correct value were Experts 8, 7, 1, 3, 6, and 4. The answer given by Expert 2 could indicate that this respondent might be considered for an inappropriate expert. For the four fixed intervals method (Figure 2h), five experts provided similar answers to those from the histogram method, while the other experts differed. Among them, Expert 2 diverged from the correct value even more. Pearson’s χ^2^ test did not reject the zero hypothesis (*p* = 0.0721): answers did not show a statistically significant difference, even though the graphic representation of the results seemed to suggest that some answers differed.

Subsequently, Expert 2 and their answers were discarded and Pearson’s χ^2^ test was applied again. The results showed that without the answers of Expert 2, the first and the fourth question again did not reject the zero hypothesis (*p* = 0.3772 and *p* = 0.5738), and the statistical test without the answers of Expert 2 for the second and the third question steadily rejected the zero hypothesis (*p* = 0.0151 and *p* = 0.00024).

**Figure 3:**
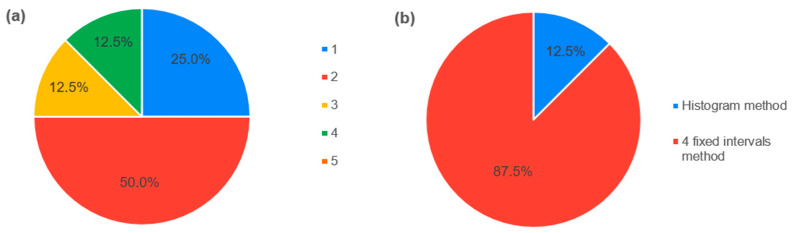
Expert’s subjective preferences. (**a**) Answers to the question about the difficulty of the study. (**b**) Answers to the question about which method is more reliable.

The next part of the questionnaire focused on the expert’s subjective preferences. Figure 3a presents the answer to the question regarding the difficulty of the study, evaluated on a scale from “1—very difficult” to “5—very easy”. Finally, all of the experts agreed that the four fixed intervals method was easier to understand, and, as for the reliability, seven experts recommended the four fixed intervals method as the more reliable method (Figure 3b).

## 4. Discussion

At present, health technology assessment has become a topical issue due to rapid advances in the medical field, efforts to improve the overall quality of healthcare, and efforts to maintain the overall costs of healthcare within reasonable limits. Health technology assessments are typically executed at the same time as (rather expensive) clinical studies. However, the results of a clinical study need not be positive for the tested technology. For medical devices in particular, there have been efforts to introduce and institutionalize early health technology assessment (eHTA) in the last years [2,4,23,24,25]. Elicitation, a strategy for filling gaps in available information about a new technology through soliciting expert opinions, is still the least described part of the eHTA methodology [1,5].

There are several elicitation methods with quite different procedures, and before launching an eHTA study, it is important to select an appropriate elicitation method in order to yield objective and reliable results. The main objective of this study, therefore, was to analyse and compare two elicitation approaches—the four fixed intervals method and the histogram method—and apply them as a pilot to the example of a new technology from the field of tissue engineering—artificial thymus [10,11,15,16].

The questionnaire was completed by all eight experts invited to participate in the survey. This can be compared with studies conducted by Grigore et al. [8] and Qi Cao et al. [26], where only some of the invited experts were able to complete the questionnaire. Their results might have been affected by the choice of experts without much experience with statistics, lacking the necessary professional qualification, or by poorly conceived questions. In our survey, we set four brief questions regarding the artificial thymus. The number of questions was deliberately limited to make sure that all of the experts would be able to fill in the entire questionnaire.

The visual and/or statistical analysis of the two elicitation methods did not unequivocally indicate which approach better reflected the real opinion of experts. Visually comparisons of the answers of experts obtained by both methods looked quite similar. The results of Pearson’s χ^2^ tests showed that the zero hypothesis could not be rejected in the first and fourth question (*p* = 0.3772 and *p* = 0.5738, respectively), i.e., we could not reject that the answers derived by both elicitation methods did not differ from each other. But the results of Pearson’s χ^2^ tests applied on the answers to the second and the third question rejected the zero hypothesis (*p* = 0.0151 and *p* = 0.00024), and the answers were statistically significantly different. A larger study with a higher number of targeted questions is needed to shed more light on the matter. However, according to Grigore et al. [8], who recommended that elicitation methods include 6 to 12 respondents, the group of eight experts was sufficient for this study.

For the first question on the issue of DiGeorge syndrome, Expert 8 was the only one whose answer using the four fixed intervals method was significantly different from the answer they provided in the histogram method. This inconsistency may be due to a misunderstanding of the elicitation methods, although this explanation was not supported by the responses to the other questions.

The statistical test for the second question (the percentage of patients with complete DiGeorge syndrome who could see improvements in their health state after an application of the artificial thymus) rejected the zero hypothesis (*p* = 0.0069), i.e., there were differences in answers provided by the two elicitation approaches. The differences might be caused by the situation, in which the experts could think things over again after they had already answered using the histogram method, and decide to change their opinion when answering using the four fixed intervals method.

Answers to the third question (possible mortality due to the application of the artificial thymus) using the histogram method showed a high level of expert uncertainty. This uncertainty might have been caused by the fact that deaths are usually linked to many factors and the experts were reluctant to form premature conclusions regarding the artificial thymus technology.

According to the answer given by Expert 2 to the fourth (“reference”) question about the current survival rate after transplantation in patients with complete DiGeorge syndrome, this respondent might be considered an inappropriate expert. However, Expert 2 had knowledge and experience corresponding to all of the requirements that were comparable to the other experts. The only thing they failed in terms of their qualification was the “reference” question. In doing so, this expert had previous experience with elicitation, but most (5 out of 8) experts had no experience with elicitation. However, they rated their statistical knowledge as basic, as did five other experts.

The limitations of the study include limited awareness of experts about the new technology in development, which is not yet known to the general public and does not have published data or evidence. The experts were introduced to a brief overview. However, it can be assumed that if experts had access to all of the details of technology development and were more familiar with the detailed results of the animal experiments, then they might have been more confident in their answers. On the other hand, the study was mainly focused on a comparison of the four fixed intervals and the histogram elicitation methods, and the selected technology was chosen only as a suitable example. One of the limiting factors of this study could also be the fact that the experts answered all the questions in the same order and always using both elicitation approaches—first the histogram method, then the four fixed intervals method. On the other hand, it can be stated that with the increasing number of questions, the certainty of answers also increased and the response time decreased, while the experts felt quite comfortable. Other limitations already discussed above were a low level of statistical knowledge of the participants, and the not yet fully standardized size of the expert group.

Concerning the artificial thymus technology as our case study, the results of the elicitation showed that experts evaluated the technology quite well and saw its potential. This suggests that the technology could attract investors, gain more support to complete its development, or be recommended for continued used in the early stages of clinical trials. Expert 1 was most pessimistic about the new technology, but they, too, eventually agreed on mortality rate with other experts when answering the third question. Because of the above limitations, it will be appropriate to carry out deeper training of experts and provide them access to all of the results of previous research on the artificial thymus. According to the eHTA procedure, the data obtained could be subsequently used to evaluate the cost-effectiveness of the technology. Such an analysis could help decide about the future of new medical technologies, in this case the artificial thymus technology, at an early stage of their development. Nevertheless, we were not seeking evidence on the artificial thymus application, but general information on the elicitation methods. Any information on artificial thymus gained was just a by-product of the study.

Future lines of research will be focused on the real application of elicitation methods within the eHTA process of new healthcare technology, where the use of numerical elicitation methods will probably be preferred, because the numerical approach was shown to be better understood and preferred by medical and non-medical experts in the field of healthcare in this research.

## 5. Conclusions

Based on the respondents’ subjective preferences, we recommend the four fixed intervals method for both medical and non-medical experts participating in healthcare research, as it is easy to understand and apply for participants without sound statistical expertise. In conclusion, before applying elicitation methods in practice, it is important to consider what technology the study will focus on, and, therefore, which experts will be invited to express their opinions.

The histogram method is more complex and harder to understand, not only because of the graphic representation, but also because of the higher number of intervals, and it is more demanding for experts than the four fixed intervals method. However, this method seems to yield more precise results thanks to its finer division of intervals. This method is probably more suitable for non-medical technically oriented experts that should be familiar with basic statistical methods. If experts’ profession or educational background do not provide a clear indication of their statistical skills, we recommend using a short questionnaire focusing on statistical abilities before the elicitation itself, which would help select suitable experts. A specific elicitation method should be chosen based on the results of such a preliminary test. At the same time, parameters such as the previous experience and statistical skills of experts, can be used together with the “reference” question for further processing of the results, such as assigning weights of expert opinions and/or subsequent aggregation.

## Data Availability

Data are contained within the article or Supplementary Material.

## Supplementary Materials

The following supporting information can be downloaded at: https://www.mdpi.com/article/10.3390/healthcare11223002/s1. S1. English translation of the questionnaire. S2. Data set.
