# Supplementary material for: Comparison of Elicitation Approaches in Early Stage HTA Applied on Artificial Thymus for Patients with DiGeorge Syndrome
Source: Healthcare (Basel). 2023 Nov 20;11(22):3002. doi: 10.3390/healthcare11223002 (PMC10671534; doi:10.3390/healthcare11223002)
Supplement: Supplementary file 1 [file healthcare-11-03002-s001.zip › healthcare-2672020-supplementary.pdf]

S1. English translation of the questionnaire

This Appendix presents an English translation of the Questionnaire that was originally distributed in Czech. The questionnaire was filled in offline, and the respondents were instructed that its submission is fully voluntary.

Questionnaire

Dear Madam/Sir,
we are presenting to you a questionnaire exploring and comparing expert opinion elicitation methods used as part of eHTA. We would greatly appreciate if you could use this questionnaire to share your expert opinion on the issues described below.

All opinions and comments gathered as part of this study will be anonymised. All information and data will be used exclusively for the study of how expert opinion elicitation methods are applied and for probability distribution within this research.

Topic

The aim of this questionnaire is to compare expert opinion elicitation methods, i.e. methods used to gather expert opinions in the form of probability distribution performed as part of an eHTA (early stage health technology assessment).

Due to the high cost of clinical assessments of health technologies an early assessment can help reduce this cost. An early stage assessment can yield valuable information for design and development of new health technologies and mitigation of risks associated with the launch of these technologies in the market.

Within eHTA, expert opinion elicitation serves primarily to add unknown parameters to new technologies. In the case of this particular study, we focus on the artificial thymus technology, which is currently being developed and tested on animals and is expected to be used in the future in the treatment of complete DiGeorge syndrome.

Complete DiGeorge syndrome is a congenital genetic defect where the thymus is completely absent. This syndrome is accompanied by a host of other conditions such as cleft palate, mental retardation, heart defects and limited kidney function. The absence of a thymus leads to an overall immunodeficiency, making the patient vulnerable to even the most banal infection. Currently the only, most successful method of treating complete DiGeorge syndrome is transplantation. However, this method comes at the risk of complications. Another disadvantage of this treatment is the fact that the best candidates for thymus donors are very young patients, whose number is very limited.

A great hope for complete DiGeorge syndrome patients is the development of an artificial thymus that could prevent these complications. Development of an artificial thymus has been explored in the field of tissue engineering, resulting in the design of “scaffolds”, i.e. special supporting structures make of biocompatible materials. These scaffolds are then planted with the patient’s own pluripotent stem cells and a new organoid is grown in vitro. The use of the patient’s own stem cells, this organoid should eliminate the risk of the patient’s body rejecting the new structure. The new thymus organoids have so far only been implanted subcutaneously in lab mice without natural thymuses. After two weeks, the organoid is colonised by new blood vessels, is able to attract T-cell precursors, followed by their maturation. Research has also focused on the effect an organoid application might have after the application of tumour cells. The result is a slowdown in the tumour growth three weeks after application. As no further results of the artificial thymus method are available as of yet we would greatly appreciate your expert opinion on the values of selected unknown parameters of the new technology based on your professional expertise and practical experience with conventional methods of treatment of patients with complete DiGeorge syndrome.

Several methods may be used to solicit expert opinions. In our study, we decided to use the histogram method and the fixed interval method. The two methods will be described in more detail in the Instructions section.

Instructions

Elicitation is a formal approach to transforming one’s knowledge into a statistical form. There are many cognitive processes that make it difficult for people to define probabilities. It is especially important to keep three types of distortion under control. One of these distortions

is anchoring – the respondent should be base his/her answer on the values stated in previous questions, instead answering each question without reference to or in the light of the previous questions. It is also important to give answers to all the questions.

This questionnaire will test both elicitation methods and help determine which one is more useful and easier to understand. Both methods will represent your subjective opinion on the issue at hand.

Histogram method

This method produces a graphic representation of the probability distribution of expert opinion. All answers will be entered into tables by means of 20 crosses. Each cross represents a 5% probability that the true value lies in the relevant interval. The sum of all crosses in one table must be 100%. By placing crosses in selected squares, you give your opinion regarding the probability and certainty of each question.

The lines in the table represent the percentage of patient – the width of the range should reflect certainty: the smaller the certainty of determining a specific patient percentage, the wider the interval.

The columns in the table represent probability. The height of a value should reflect the probability of the answer: the higher the probability value of the determined patient percentage, the higher the space above the value. To clarify, we offer an example of a question and a possible answer.

Question: How many patients own a pet? Answer: I think that there is a 100% probability that 50% patients own a pet. I am, therefore, certain that 50% patients own a pet. This form of answer represents one of the possible extremes. In practice, however, such answers are rather rare.

Pravděpodobnost %

100										X										
95										X										
90										X										
85										X										
80										X										
75										X										
70										X										
65										X										
60										X										
55										X										
50										X										
45										X										
40										X										
35										X										
30										X										
25										X										
20										X										
15										X										
10										X										
5										X										
	5	10	15	20	25	30	35	40	45	50	55	60	65	70	75	80	85	90	95	100

Pacienti %

Another example of a potential extreme answer: I am not sure how many patients have pets, so the probability is the same for all.

Pravděpodobnost v %

100																				
95																				
90																				
85																				
80																				
75																				
70																				
65																				
60																				
55																				
50																				
45																				
40																				
35																				
30																				
25																				
20																				
15																				
10																				
5	X	X	X	X	X	X	X	X	X	X	X	X	X	X	X	X	X	X	X	X
	5	10	15	20	25	30	35	40	45	50	55	60	65	70	75	80	85	90	95	100

Pacienti %

Fixed interval method

This is the other method that will serve to determine expert opinion. However, unlike the histogram method, it does not involve a graphic representation but only numerical data. In this case, a specific probability value in % is assigned to each interval. The sum of the probability values in a question must always be 100%. Unlike in the histogram method, the respondent does not have to consider the certainty of answers here. Once again, we give examples of possible answers to one question.

Question: How many patients own a pet? Answer: I think there is a 55% probability that 25% to 50% of patients have a pet. I think it's less probable that 50% to 75% patients or only 0% to 25% patients have a pet, so I give 25% and 20% to these intervals, respectively. And I don't think at all that 75% to 100% patients have a pet, so I give a 0% probability to this interval.

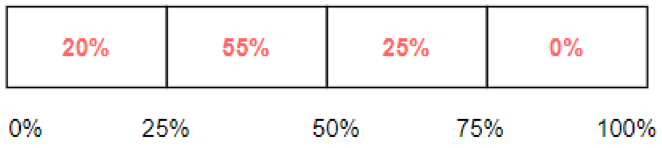

Here is your opportunity to try answering the same question: how many patients do you think have a pet?

Answer in the histogram method:

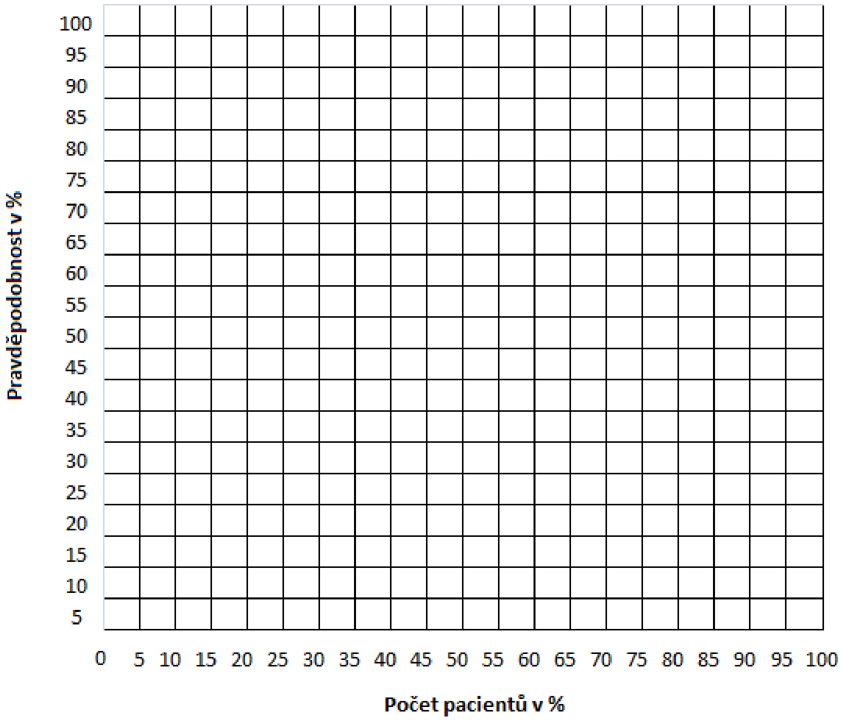

Answer in the fixed interval method:

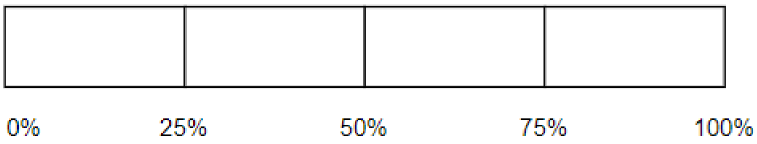

Expert questions

The following questions will be answered on the same principles as the example questions in the Instructions section above. Give two answers to each question – one using the histogram method and one using the fixed interval method.

1. What percentage of patients with complete DiGeorge syndrome would be suitable candidates for artificial thymus treatment?

Enter your subjective opinion into the following table using the histogram method as described above in the Instructions section (20 crosses).

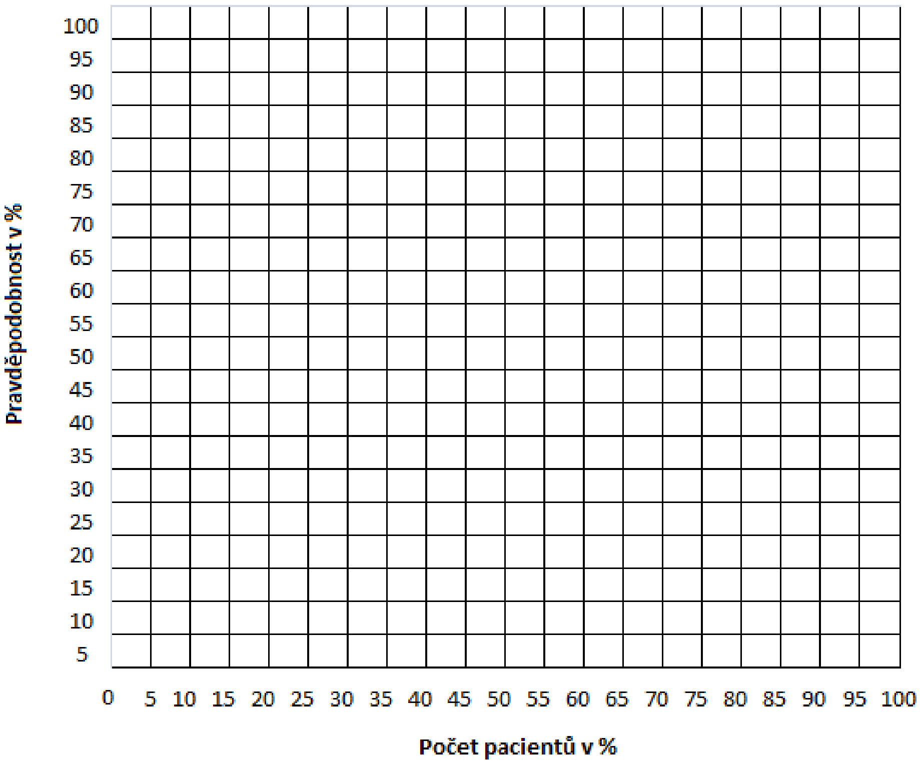

Enter your subjective opinion into the following table using the fixed interval method as described above in the Instructions section.

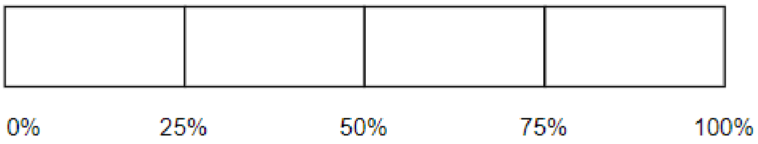

2. In your opinion, what percentage of patients with complete DiGeorge syndrome could see an improvement in their health condition after application of an artificial thymus?

Enter your subjective opinion into the following table using the histogram method as described above in the Instructions section (20 crosses).

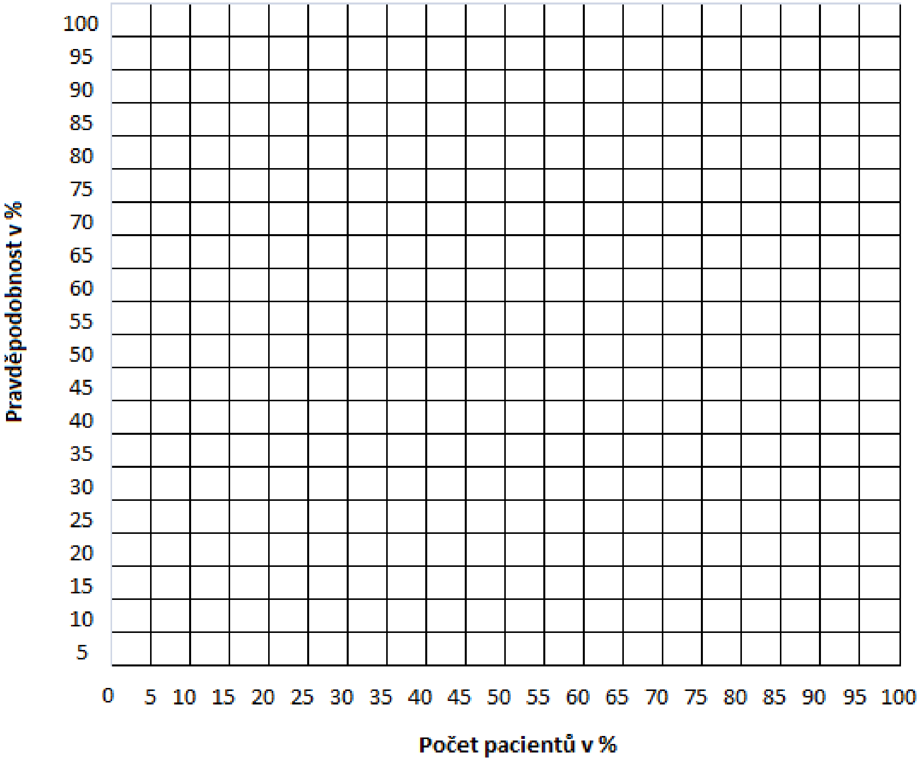

Enter your subjective opinion into the following table using the fixed interval method as described above in the Instructions section.

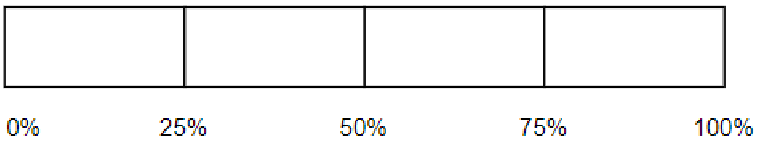

3. In your opinion, what percentage of patients with complete DiGeorge syndrome could die due to application of an artificial thymus?

Enter your subjective opinion into the following table using the histogram method a described above in the Instructions section (20 crosses).

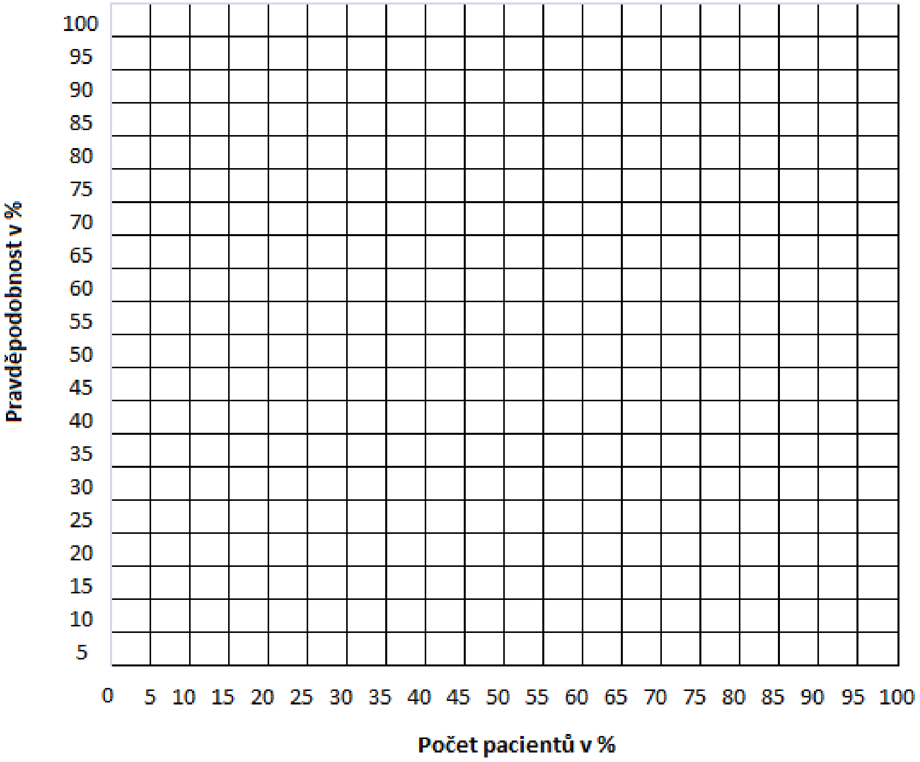

Enter your subjective opinion into the following table using the fixed interval method as described above in the Instructions section.

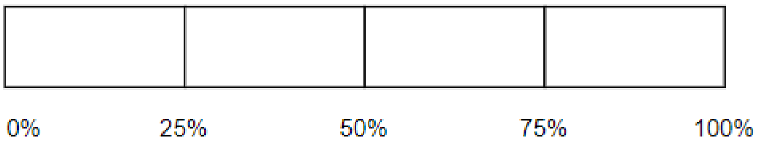

4. What is the current survival rate of patients with complete DiGeorge syndrome after transplantation?

Enter your subjective opinion into the following table using the histogram method a described above in the Instructions section (20 crosses).

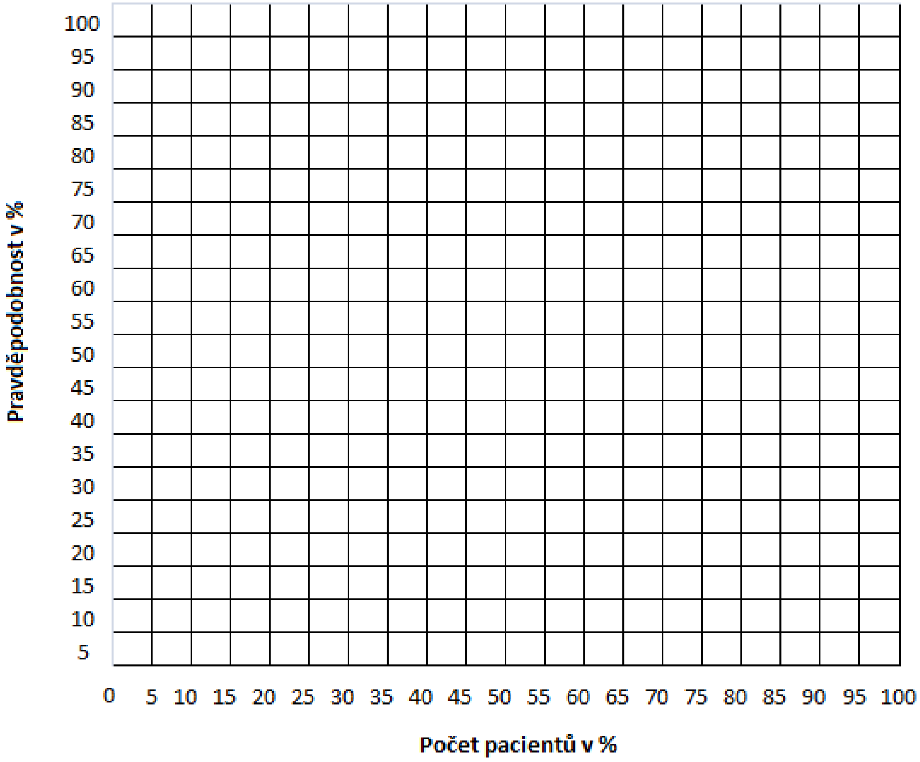

Enter your subjective opinion into the following table using the fixed interval method as described above in the Instructions section.

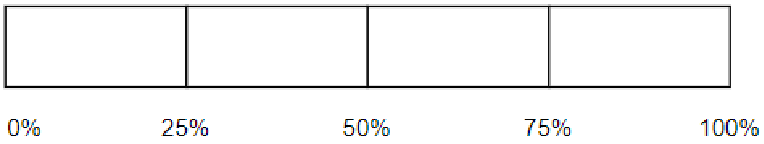

Additional questions

This part of the questionnaire includes additional questions related to this study and your opinion on the elicitation methods used in this study.

1. Have you ever participated in any research formally assessing expert opinion? (put a cross under your answer)

YES	NO

2. How would you assess your own statistical skills? (put a cross under your answer)

Basic	Good	Excellent

3. How difficult was it for you to complete this questionnaire. Give an answer on a scale from 1 to 5 where 1 means "very difficult to complete" and 5 means "very easy to complete". (put a cross under your answer)

1	2	3	4	5

4. Which method was easier for you to understand? (put a cross under your answer)

Histogram method	Fixed interval method

5. Which method do you think is more reliable? (put a cross under your answer)

Histogram method	Fixed interval method

Thank you for taking the time to fill this questionnaire.

S2. Data set

The following table contains all individual answers to Questions 1-4. All data concerning the additional questions are included directly in the paper.

Histogram method	Intervals	Expert 1	Expert 2	Expert 3	Expert 4	Expert 5	Expert 6	Expert 7	Expert 8
1st Question	0-5	0.00%	0.00%	40.00%	0.00%	0.00%	0.00%	0.00%	0.00%
	5-10	55.00%	0.00%	20.00%	0.00%	0.00%	0.00%	0.00%	0.00%
	10-15	25.00%	0.00%	15.00%	0.00%	0.00%	0.00%	0.00%	0.00%
	15-20	20.00%	0.00%	10.00%	0.00%	0.00%	0.00%	0.00%	0.00%
	20-25	0.00%	0.00%	10.00%	0.00%	0.00%	0.00%	0.00%	0.00%
	25-30	0.00%	0.00%	5.00%	0.00%	0.00%	0.00%	0.00%	0.00%
	30-35	0.00%	0.00%	0.00%	0.00%	0.00%	0.00%	0.00%	0.00%
	35-40	0.00%	0.00%	0.00%	0.00%	0.00%	5.00%	0.00%	0.00%
	40-45	0.00%	0.00%	0.00%	0.00%	0.00%	15.00%	15.00%	0.00%
	45-50	0.00%	0.00%	0.00%	0.00%	0.00%	25.00%	40.00%	5.00%
	50-55	0.00%	0.00%	0.00%	0.00%	0.00%	35.00%	20.00%	5.00%
	55-60	0.00%	0.00%	0.00%	0.00%	5.00%	10.00%	15.00%	5.00%
	60-65	0.00%	0.00%	0.00%	10.00%	10.00%	5.00%	10.00%	5.00%
	65-70	0.00%	0.00%	0.00%	20.00%	40.00%	5.00%	0.00%	10.00%
	70-75	0.00%	0.00%	0.00%	40.00%	25.00%	0.00%	0.00%	20.00%
	75-80	0.00%	5.00%	0.00%	20.00%	15.00%	0.00%	0.00%	30.00%
	80-85	0.00%	10.00%	0.00%	10.00%	5.00%	0.00%	0.00%	10.00%
	85-90	0.00%	80.00%	0.00%	0.00%	0.00%	0.00%	0.00%	5.00%
	90-95	0.00%	5.00%	0.00%	0.00%	0.00%	0.00%	0.00%	5.00%
	95-100	0.00%	0.00%	0.00%	0.00%	0.00%	0.00%	0.00%	0.00%
Fixed interval method	Intervals	Expert 1	Expert 2	Expert 3	Expert 4	Expert 5	Expert 6	Expert 7	Expert 8
	0-25	70.00%	0.00%	85.00%	0.00%	0.00%	0.00%	0.00%	50.00%

	**25-50**	**20.00%**	**0.00%**	**12.00%**	**20.00%**	**20.00%**	**30.00%**	**20.00%**	**40.00%**
	**50-75**	**10.00%**	**0.00%**	**3.00%**	**60.00%**	**65.00%**	**40.00%**	**60.00%**	**10.00%**
	**75-100**	**0.00%**	**100.00%**	**0.00%**	**20.00%**	**15.00%**	**30.00%**	**20.00%**	**0.00%**
**Histogram method**	**Intervals**	**Expert 1**	**Expert 2**	**Expert 3**	**Expert 4**	**Expert 5**	**Expert 6**	**Expert 7**	**Expert 8**
**2nd Question**	**0-5**	**0.00%**	**0.00%**	**0.00%**	**0.00%**	**0.00%**	**0.00%**	**0.00%**	**0.00%**
	**5-10**	**0.00%**	**0.00%**	**0.00%**	**0.00%**	**0.00%**	**0.00%**	**0.00%**	**0.00%**
	**10-15**	**0.00%**	**0.00%**	**0.00%**	**0.00%**	**0.00%**	**0.00%**	**0.00%**	**0.00%**
	**15-20**	**0.00%**	**0.00%**	**0.00%**	**0.00%**	**0.00%**	**0.00%**	**0.00%**	**0.00%**
	**20-25**	**10.00%**	**0.00%**	**0.00%**	**0.00%**	**0.00%**	**0.00%**	**0.00%**	**0.00%**
	**25-30**	**40.00%**	**0.00%**	**0.00%**	**0.00%**	**0.00%**	**0.00%**	**0.00%**	**0.00%**
	**30-35**	**20.00%**	**0.00%**	**0.00%**	**0.00%**	**0.00%**	**0.00%**	**0.00%**	**0.00%**
	**35-40**	**15.00%**	**0.00%**	**0.00%**	**0.00%**	**0.00%**	**0.00%**	**0.00%**	**0.00%**
	**40-45**	**10.00%**	**0.00%**	**0.00%**	**0.00%**	**0.00%**	**0.00%**	**0.00%**	**0.00%**
	**45-50**	**5.00%**	**0.00%**	**0.00%**	**0.00%**	**0.00%**	**0.00%**	**0.00%**	**0.00%**
	**50-55**	**0.00%**	**0.00%**	**5.00%**	**0.00%**	**0.00%**	**5.00%**	**0.00%**	**0.00%**
	**55-60**	**0.00%**	**70.00%**	**5.00%**	**0.00%**	**0.00%**	**10.00%**	**5.00%**	**0.00%**
	**60-65**	**0.00%**	**20.00%**	**25.00%**	**0.00%**	**0.00%**	**15.00%**	**10.00%**	**0.00%**
	**65-70**	**0.00%**	**5.00%**	**50.00%**	**0.00%**	**0.00%**	**20.00%**	**15.00%**	**45.00%**
	**70-75**	**0.00%**	**5.00%**	**10.00%**	**0.00%**	**0.00%**	**40.00%**	**35.00%**	**25.00%**
	**75-80**	**0.00%**	**0.00%**	**5.00%**	**0.00%**	**0.00%**	**5.00%**	**15.00%**	**15.00%**
	**80-85**	**0.00%**	**0.00%**	**0.00%**	**10.00%**	**30.00%**	**5.00%**	**10.00%**	**10.00%**
	**85-90**	**0.00%**	**0.00%**	**0.00%**	**15.00%**	**45.00%**	**0.00%**	**5.00%**	**5.00%**
	**90-95**	**0.00%**	**0.00%**	**0.00%**	**25.00%**	**20.00%**	**0.00%**	**5.00%**	**0.00%**
	**95-100**	**0.00%**	**0.00%**	**0.00%**	**50.00%**	**5.00%**	**0.00%**	**0.00%**	**0.00%**
**Fixed interval method**	**Intervals**	**Expert 1**	**Expert 2**	**Expert 3**	**Expert 4**	**Expert 5**	**Expert 6**	**Expert 7**	**Expert 8**
	**0-25**	**20.00%**	**0.00%**	**2.00%**	**0.00%**	**0.00%**	**10.00%**	**0.00%**	**5.00%**
	**25-50**	**50.00%**	**15.00%**	**6.00%**	**0.00%**	**0.00%**	**50.00%**	**30.00%**	**35.00%**
	**50-75**	**20.00%**	**70.00%**	**77.00%**	**20.00%**	**20.00%**	**40.00%**	**50.00%**	**50.00%**

	75-100	10.00%	15.00%	15.00%	80.00%	80.00%	0.00%	20.00%	10.00%
**Histogram method**	**Intervals**	**Expert 1**	**Expert 2**	**Expert 3**	**Expert 4**	**Expert 5**	**Expert 6**	**Expert 7**	**Expert 8**
3rd Question	0-5	0.00%	5.00%	30.00%	20.00%	0.00%	0.00%	5.00%	0.00%
	5-10	0.00%	10.00%	25.00%	20.00%	0.00%	0.00%	5.00%	0.00%
	10-15	0.00%	20.00%	15.00%	20.00%	5.00%	0.00%	5.00%	30.00%
	15-20	0.00%	30.00%	10.00%	20.00%	5.00%	0.00%	10.00%	40.00%
	20-25	5.00%	20.00%	5.00%	20.00%	5.00%	0.00%	15.00%	30.00%
	25-30	10.00%	10.00%	5.00%	0.00%	5.00%	5.00%	30.00%	0.00%
	30-35	15.00%	5.00%	5.00%	0.00%	15.00%	5.00%	10.00%	0.00%
	35-40	50.00%	0.00%	5.00%	0.00%	40.00%	10.00%	10.00%	0.00%
	40-45	15.00%	0.00%	0.00%	0.00%	10.00%	15.00%	5.00%	0.00%
	45-50	5.00%	0.00%	0.00%	0.00%	5.00%	20.00%	5.00%	0.00%
	50-55	0.00%	0.00%	0.00%	0.00%	5.00%	30.00%	0.00%	0.00%
	55-60	0.00%	0.00%	0.00%	0.00%	5.00%	10.00%	0.00%	0.00%
	60-65	0.00%	0.00%	0.00%	0.00%	0.00%	5.00%	0.00%	0.00%
	65-70	0.00%	0.00%	0.00%	0.00%	0.00%	0.00%	0.00%	0.00%
	70-75	0.00%	0.00%	0.00%	0.00%	0.00%	0.00%	0.00%	0.00%
	75-80	0.00%	0.00%	0.00%	0.00%	0.00%	0.00%	0.00%	0.00%
	80-85	0.00%	0.00%	0.00%	0.00%	0.00%	0.00%	0.00%	0.00%
	85-90	0.00%	0.00%	0.00%	0.00%	0.00%	0.00%	0.00%	0.00%
	90-95	0.00%	0.00%	0.00%	0.00%	0.00%	0.00%	0.00%	0.00%
	95-100	0.00%	0.00%	0.00%	0.00%	0.00%	0.00%	0.00%	0.00%
**Fixed interval method**	**Intervals**	**Expert 1**	**Expert 2**	**Expert 3**	**Expert 4**	**Expert 5**	**Expert 6**	**Expert 7**	**Expert 8**
	0-25	20.00%	75.00%	80.00%	100.00%	40.00%	20.00%	20.00%	85.00%
	25-50	30.00%	20.00%	15.00%	0.00%	40.00%	50.00%	40.00%	10.00%
	50-75	20.00%	5.00%	5.00%	0.00%	20.00%	20.00%	20.00%	5.00%
	75-100	30.00%	0.00%	0.00%	0.00%	0.00%	10.00%	20.00%	0.00%

Histogram method	Intervals	Expert 1	Expert 2	Expert 3	Expert 4	Expert 5	Expert 6	Expert 7	Expert 8
4th Question	0-5	0.00%	0.00%	0.00%	0.00%	0.00%	0.00%	0.00%	0.00%
	5-10	0.00%	0.00%	0.00%	0.00%	0.00%	0.00%	0.00%	0.00%
	10-15	0.00%	0.00%	0.00%	0.00%	0.00%	0.00%	0.00%	0.00%
	15-20	0.00%	0.00%	0.00%	0.00%	0.00%	0.00%	0.00%	0.00%
	20-25	0.00%	5.00%	0.00%	0.00%	0.00%	0.00%	0.00%	0.00%
	25-30	0.00%	10.00%	0.00%	0.00%	0.00%	0.00%	0.00%	0.00%
	30-35	0.00%	10.00%	0.00%	0.00%	0.00%	0.00%	0.00%	0.00%
	35-40	0.00%	15.00%	0.00%	0.00%	0.00%	0.00%	0.00%	0.00%
	40-45	0.00%	30.00%	0.00%	0.00%	0.00%	0.00%	0.00%	0.00%
	45-50	0.00%	10.00%	5.00%	0.00%	0.00%	0.00%	0.00%	0.00%
	50-55	0.00%	10.00%	5.00%	0.00%	0.00%	0.00%	5.00%	0.00%
	55-60	0.00%	5.00%	5.00%	0.00%	0.00%	5.00%	15.00%	0.00%
	60-65	0.00%	5.00%	5.00%	0.00%	0.00%	5.00%	20.00%	0.00%
	65-70	0.00%	0.00%	10.00%	0.00%	15.00%	5.00%	55.00%	0.00%
	70-75	0.00%	0.00%	30.00%	0.00%	55.00%	15.00%	5.00%	60.00%
	75-80	60.00%	0.00%	15.00%	0.00%	15.00%	25.00%	0.00%	20.00%
	80-85	25.00%	0.00%	15.00%	10.00%	15.00%	45.00%	0.00%	10.00%
	85-90	15.00%	0.00%	5.00%	80.00%	0.00%	0.00%	0.00%	10.00%
	90-95	0.00%	0.00%	5.00%	10.00%	0.00%	0.00%	0.00%	0.00%
	95-100	0.00%	0.00%	0.00%	0.00%	0.00%	0.00%	0.00%	0.00%
Fixed interval method	Intervals	Expert 1	Expert 2	Expert 3	Expert 4	Expert 5	Expert 6	Expert 7	Expert 8
	0-25	0.00%	30.00%	5.00%	0.00%	10.00%	0.00%	0.00%	0.00%
	25-50	10.00%	30.00%	20.00%	0.00%	10.00%	0.00%	20.00%	0.00%
	50-75	20.00%	30.00%	55.00%	0.00%	40.00%	50.00%	60.00%	80.00%
	75-100	70.00%	10.00%	20.00%	100.00%	40.00%	50.00%	20.00%	20.00%
